# Involvement of thrombopoietin in the development of organ injury in a mouse model of cecal ligation and puncture-induced sepsis

**DOI:** 10.1186/cc11742

**Published:** 2012-11-14

**Authors:** E Greco, A Cuccurullo, B Laface, D Gallo, O Bosco, P De Giuli, E Lupia, G Montrucchio

**Affiliations:** 1 University of Turin, Italy

## Background

Sepsis-induced organ damage is a leading cause of death in critically ill patients. Thrombopoietin (TPO) is a humoral growth factor mainly involved in regulation of platelet number and function. High circulating levels of TPO are detectable in septic adults and children and are related to sepsis severity. We have previously shown a correlation between TPO levels and platelet activation in septic burned patients, where circulating activated platelets may cause microthrombotic events that lead to organ damage. Our aim was to evaluate the contribution of TPO in organ injury in a murine model of polymicrobial sepsis. To this end, we synthesized and used a chimeric fusion protein, named mouse TPO Receptor-Maltose Binding Protein (mTPOR-MBP), able to inhibit TPO biological activity.

## Methods

Male C57BL/6 mice were randomized to cecal ligation and puncture-induced sepsis (CLP) or to laparotomy (sham) surgery. CLP mice received 40 μg mTPO-MBP or sterile saline 10 minutes after surgery. Immediately after and 6 hours after surgery all animals received 0.08 mg/kg buprenorfin in 1.5 ml sterile saline subcutaneously. After 18 hours blood was collected from the cava vein and used for cell count, flow cytometric analysis of leukocyte-platelet interaction and to obtain plasma. Plasma TPO levels were determined by ELISA. The lung and liver were excised and fixed in 4% paraformaldehyde solution. An expert pathologist blinded to experimental groups quantified organ damage.

## Results

Plasma TPO levels were significantly higher in septic mice (nine mice in each group). Leukocyte and platelet counts did not significantly differ in the CLP group treated with mTPOR-MBP compared with the CLP control group. In contrast, the percentage of monocyte-platelet aggregates, a marker of platelet activation, was significantly reduced after treatment with mTPOR-MBP. Moreover, TPO blockade by mTPOR-MBP administration induced a significant reduction of histological alterations in the lung, as evaluated by neutrophil infiltration and thickening of the alveolar-capillary membrane, and liver tissue samples, as evaluated by the number of microabscesses.

## Conclusion

Increased circulating levels of TPO during experimental sepsis may have a role in the development of organ damage. Inhibition of TPO biological activity may represent a novel promising therapeutic approach to prevent organ failure in severe sepsis.

